# Exploring the challenges faced by generic version of complex drugs: a scoping review

**DOI:** 10.1186/s13643-025-02931-y

**Published:** 2025-09-29

**Authors:** Amatha Sreedevi, Elstin Anbu Raj, D. Sreedhar, Virendra S. Ligade

**Affiliations:** 1 Department of Pharmaceutical Regulatory Affairs and Management, Manipal College of Pharmaceutical Sciences, Manipal Academy of Higher Education, Manipal, Karnataka 576104 India; 2 Center for Evidence-Informed Decision Making, Prasanna School of Public Health, Manipal Academy of Higher Education, Manipal, Karnataka 576104 India

**Keywords:** Complex generics, Hybrid drugs, Critical quality attributes, Quality and safety, Hurdles

## Abstract

**Background:**

Complex generics can be defined by their complex active pharmaceutical ingredients (APIs), formulations, dosage forms, routes of administration, and drug device combinations which significantly pose challenges in scientific advancement and regulatory approvals. The present review aims to investigate, identify, and classify the critical challenges faced by different stakeholders and the strategies adopted to address these challenges across the developmental stage till the post-approval phases of complex generics.

**Method:**

We followed the Preferred Reporting Items for the Scoping Reviews Extension for Systematic Reviews and Meta-Analyses (PRISMA-ScR) guidelines for the systematic search conducted across various databases such as PubMed, EMBASE, Scopus, and Web of Science (January 1, 2014, to August 22, 2024). English language articles that addressed various challenges as well as innovative strategic approaches were included in the study. Two authors independently reviewed the retrieved papers to reduce the possibility of bias. The screening was performed based on predetermined inclusion and exclusion criteria.

**Results:**

A total of 1718 studies were retrieved from the database searches, from which 24 met the eligibility criteria. The selected articles were from the USA, the European Union, India, Russia, Taiwan, China, and Malaysia. The formulation challenges (17 articles), analytical challenges (19 articles), clinical challenges (18 articles), critical process parameter-related challenges (17 articles), critical quality attribute-related challenges (19 articles), and regulatory challenges (21 articles) were the major challenges of complex generics. The use of advanced sophisticated analytical equipment as well as orthogonal bioanalytical testing, the implementation of a dynamic regulatory cross-checking system, the development and use of machine learning and artificial intelligence tools, and the development of quality-by-design approaches and models have been recognized as the best methods for addressing these challenges.

**Conclusion:**

This review addresses critical gap by providing a systematic summary of stakeholder-reported barriers and novel mitigation approaches in the complex generics domain. It highlights the urgent need for harmonized global regulatory guidelines, advance analytical tools, and strategic stakeholder collaboration to hasten the development and availability of high-quality complex generics.

**Scoping review registration:**

The review was not registered.

**Supplementary Information:**

The online version contains supplementary material available at 10.1186/s13643-025-02931-y.

## Introduction

Over the past few decades, pharmaceutical companies have been driven by advanced drug discovery and have employed innovative drug development and analytical techniques [1]. Along with this advance, the growth of generic companies has drastically increased. The growth of simple generic drugs has steadily increased, and generic versions of these complex drugs are fewer in number, which again garners more attention from generic companies as well as more money from the public [2]. Currently, many branded drugs do not have a generic competitor on the market even after product-specific guidelines are published. The likelihood of abbreviated new drug application (ANDA) lies in the complexity of innovator products because of development challenges and hurdles in establishing bioequivalence [3]. Researchers and regulatory bodies are paying significant attention to developing cost-effective medicaments for these complex, innovative, branded drugs. These cost-effective generic versions of complex drugs are called complex generics, hybrid medicines, specialty medicines, value-added medicines, or improved therapeutic entities [4]. Complex generics are products that have complexity in establishing bioequivalence (BE). According to the US Food and Drug Administration (FDA), “Complex generics are those drug products generally include products with complex active ingredients, complex formulations, complex routes of delivery, complex dosage forms OR complex drug-device combination products OR other products where complexity or uncertainty concerning the approval pathway or possible alternative approach would benefit from early scientific engagement” [5]. Complex generics act as a booster in developing countries to address the unmet needs of patients for advanced drugs at a relatively low rate without compromising quality [4]. However, several distinct challenges must be overcome to successfully develop, approve, and market complex generics. These obstacles come from technological advances in characterization, scientific findings, advanced manufacturing processes, clinical studies, laws and regulations, and healthcare sectors [6].

Complex generics have unique challenges that separate them from traditional generics. A few years ago, due to a lack of specific regulatory guidance, arguments among the ANDA applicant and regulatory agency were often held. These complications make the journey toward marketplaces quite challenging until patient complaints occur because they frequently result in additional development costs, longer timeframes, and a greater chance of rejection [7]. A standardized BE assessment approach has been established to help generic pharmaceutical companies file more acceptable ANDA, standardize the review procedure, and enhance the effectiveness and quality of the review [8].

To guarantee the quality, efficacy, and safety of complex generics, regulatory organizations of regulated markets such as the European Medicines Agency (EMA), the US FDA, Japan (Ministry of Health, Labor, and Welfare), and Health Canada have set strict requirements. However, the primary challenge for pharmaceutical companies seeking to enter international markets frequently involves the lack of uniformity in regulatory standards across different regulatory bodies, which highlights the need for harmonized guidelines [9]. To obtain information ahead of the manufacturing process, the FDA encourages innovative strategies and exchanges between pharmaceutical companies and the regulatory body, such as controlled correspondence [10], pre-ANDA meetings [11], and product-specific guidelines for specific products [12]. According to industry viewpoints, manufacturing hurdles are more common than legal and regulatory issues. These difficulties might result in oligopolistic market dynamics since they involve significant startup and investment costs and specialized manufacturing knowledge that only large and/or established companies may possess [13]. In addition, challenges that arise throughout the growth of complex generics include robust intellectual property restrictions, citizen petitions, facility complications, lack of literature, uncertainty regarding bioequivalence/clinical trial approaches, structural characterization, device uniformity, stability of formulations by regulatory standards, and the establishment of in vitro in vivo (IVIVC) correlation [14]. Complex injectables, drug/device combination drugs, and topical and transdermal delivery system (TDS) are examples of products with particular technical difficulties that present unique obstacles [15]. Even though these products have the potential to lower healthcare costs and increase accessibility, their adoption may be hindered by a lack of solid empirical data and educational opportunities that satisfy both patient and healthcare provider fears. In their study on the need for educational initiatives on complex generics, Stern et al. conclude that the Center for Research on Complex Generics (CRCG) should propose some potential approaches, such as workshops on therapeutic equality, modeling approaches, and awareness campaigns, to overcome challenges and enhance patient accessibility [16].

Despite these difficulties, it is impossible to underestimate the potential advantages of complex generics. Complex generics provide an opportunity to improve patient availability of life-saving treatments and reduce the financial burden on medical facilities by providing lower cost substitutes for expensive innovative medicines [17]. Even though it is complicated, there is a 40–50% reduction in price compared with branded drugs. Compared with small-molecule generics (simple generics), complex generics are expensive because of their complexity [13].

### Current research in the area

The FDA granted the University of Michigan and the University of Maryland a 5-year grant on August 1, 2020, to set up a CRCG. With the goal of supporting the FDA’s objective of expanding the supply of safe and efficient generic pharmaceuticals, the center sought to strengthen research partnerships with the pharmaceutical industry. The FDA, the generics industry, and interested parties work together to conduct research, provide training, and share resources to achieve this aim [18]. “Mitigation of nitrosamine formation in solid dosage form through formulation” is one of the most popular collaborative works, which led the FDA to issue the guidance “Control of nitrosamine impurities in human drugs” in 2020 [19]. Some of the recent collaborative work on the CRCG is given below in Table 1 [20].

**Table 1:** Recent collaborative work related to complex generics of CRCG

Title	Aim
Toolkit to assess adhesion performance of topical and transdermal delivery system in vitro	To cover a wide range of key developmental issues, failure modes, and mitigation strategies to overcome adhesion failures in TDS
Reverse engineering of Invega Sustenna® (paliperidome palmitate suspension)	To establish a systematic quality characteristics assessment for Invega Sustenna®, including particle size distribution, particle morphology, thermal characteristics, crystalline properties, in vitro dissolution kinetics, and in vivo pharmacokinetics
Reverse Engineering, IVR, and small-scale manufacturing of ONIVYDE™ (irinotecan liposome injection)	Identified the need for research focused on “reverse engineering” of complex generic products and in vitro release (IVR) methodologies for liposomal products due to inherent difficulties in validating IVR methodologies and challenges in showing their discriminative abilities between batches of liposomes at various stages of the development cycle
Scientific challenges and opportunities in the development of complex generics	To understand the product portfolio of companies developing generic drugs and future considerations and opportunities in complex generic drug development
Best practices and standards in nanotechnology	To probe and update the current and upcoming needs and prioritize the development of standard analytical methods for physicochemical characterization, quality, and equivalence assessment of products that contain nanomaterials

### Why is it important to do this review?

Since our topic is relatively new, it is also a vast one. A scoping review was selected because it is a more appropriate approach to accomplish our research goal. Scoping reviews are carried out to map out the available literature on a specific field of study that has not already been reviewed, offering a chance to identify significant concepts, gaps in knowledge, and crucial sources and various kinds of findings to guide researchers, manufacturing companies, policymakers, and healthcare providers [21]. By reviewing the literature, independent of its design, this study sought to identify the challenges associated with various developmental stages of complex generics and the strategies to overcome them.

#### Novelty and relevance

To the best of our knowledge, this is the first scoping review that uses an evidence synthesis framework to thoroughly map the different barriers and solutions associated with complex generics throughout the development process, from formulation and analytical difficulties to regulatory and market access barriers. Although regulatory pathways or case studies of specific products could have been included in our prior papers or discussions, those were not systematic evidence syntheses that adhered to tried-and-true scoping review techniques like PRISMA-ScR. However, by integrating literature from both emerging and regulated markets and classifying barriers according to technical domains, stakeholder groups, and product categories, this article provides a thorough, multifaceted landscape analysis. This allows for a more thorough, broadly relevant, and practical understanding of complex generics.

#### Review questions

Since the scoping review problem is continually evolving in nature [22, 23], the following specific questions were proposed:

1. What challenges are faced during each stage of complex generics, from development to post-global market surveillance?
2. What knowledge gaps are identified, and how can we overcome them to achieve exponential future growth of complex generics?

The problem (P) in this study comprised all the articles that discussed the boundaries, challenges, or difficulties of bringing up complex generics in health systems. The gradual rise of complex generics was the concept (C), and the context included global pharmaceutical industry, regulatory bodies, health organizations, and academic research centers that rely on data to make decisions.

## Methods

The scoping review, which offered extensive details on a particular issue and in a specific context, is the best method for outlining the wide variety of available evidence. Additionally, this methodology made it possible to identify knowledge gaps. This decision supported a scoping review [24]. The review was conducted in adherence to the five steps of Arksey and O’Malley’s [25] scoping review framework, which included (a) formulating the research question; (b) finding significant studies; (c) choosing studies; (d) extracting and charting the data; and (e) compiling, summing, and reporting the findings [26]. The review was undertaken between May and August 2024. Before starting the review process, a rough protocol was prepared for our reference but not registered officially. Scoping reviews were conducted via the Joanna Briggs Institute (JBI) Evidence Synthesis guidance sheet to guarantee systematic and reproducible work [21]. The Preferred Reporting Items for Systematic Reviews and Meta-analyses extension for Scoping Reviews (PRISMA-ScR) checklist [27] was followed for reporting the review.

### Requirements for studies to be eligible for this review

The PCC (problem, concept, and context) framework was used for defining the research question for the current review. The studies assessing various complex drug classes and associated challenges as the subject matter problem were considered for the review. This research aimed to examine and identify the main problems that had arisen throughout development, obtain approval, and market authorization for complex generics, as well as potential solutions implemented by different stakeholders. Articles addressing the influence of complex generics in a global setting and the application of novel approaches to these problems were included within the source in this context. English-language publications were included, whereas contents written in different languages were omitted. As complex generics have become increasingly popular in the last 10 years, the database search was conducted between January 1, 2014, and August 22, 2024. We included all the main original research designs to ensure that our search was sufficiently broad, including the many stakeholder-reported challenges of complex generics. Any relevant review articles were also considered for inclusion focusing on the research question. However, considering that these designs were unlikely to convey our final result of interest, protocols, commentaries, books, book chapters, reports, editorials, or letters were excluded.

### Selection of information sources

The study team developed a thorough search strategy in collaboration with a scoping and systematic review consultant. As suggested by Peters et al., the search approach was executed in two phases. A preliminary search was conducted in PubMed to retrieve the words used in the title, abstract, and index terms used to characterize the articles on the subject. Medical Subject Headings (MeSH) were employed for centralized domains. Finally, a thorough search strategy for PubMed was developed using all identified keywords and phrases. For each database in our review, the definitive PubMed search approach was modified as necessary via a polyglot search translator [28]. The electronic databases included were Web of Science, Embase, Scopus, and PubMed. For a brief evidence assessment, the gray literature was excluded because conducting a thorough search could take much time, yielded very few relevant results, and had poor repeatability [29]. After the scoping review’s viability and effectiveness were evaluated, the four above databases were chosen for exploration in this process [30]. The full search strategy is outlined in Supplementary File 2.

### Screening and selection

All the articles retrieved from the search databases were imported to Rayyan.ai [31]. After the de-duplication process, the articles were screened following a two-step process by two reviewers in duplicate. An initial phase of title-abstract screening was followed by full-text screening. In case, any conflicts between the reviewers were solved through discussion or in consultation with the third reviewer. The inclusion and exclusion criteria were used at both levels of screening. The individual steps in screening were described using PRISMA flow chart. The whole screening process was done by August 2024.

### Data extraction

A data collection spreadsheet intended to collect all pertinent data from various study designs to satisfy the study’s goals and research questions was developed and pilot-tested. The data extraction form was developed using Microsoft Excel. Two reviewers were involved in data extraction, and in case of any conflicts, third reviewer was consulted for making decisions. Author name, year, country, study objective, complex generic type, difficulties encountered, and techniques identified were among the information that was retrieved.

### Data analysis, presentation, and dissemination

**Fig. 1:**
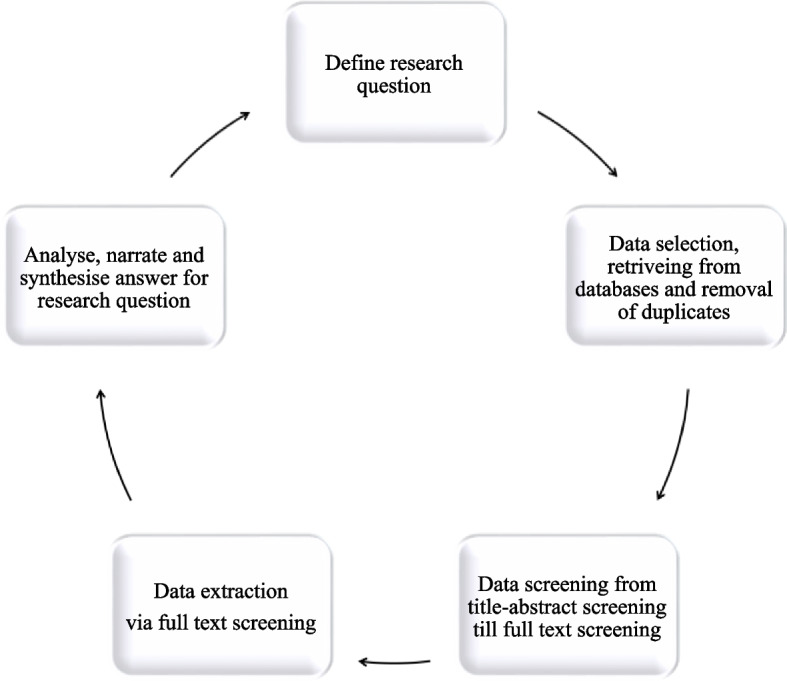
Methodological framework

Charting the data according to the Arksey and O’Malley method protocol is what this stage is known as [22]. The process of arranging, sketching, and sifting elements according to their essential properties is known as charting [26]. We reported study selection and inclusion in a PRISMA chart [32] following the PRIMSA-ScR scoping review extension guidance. The data obtained from the extraction sheet were utilized for data analysis. The analysis included both quantitative and qualitative approaches based on the extracted data. A narrative synthesis method was adopted for reporting the results based on the type of complex generics and various challenges identified through the review. A visual flow diagram of the methodological framework (Fig. 1) is advisable for clarity.

## Results

**Fig. 2:**
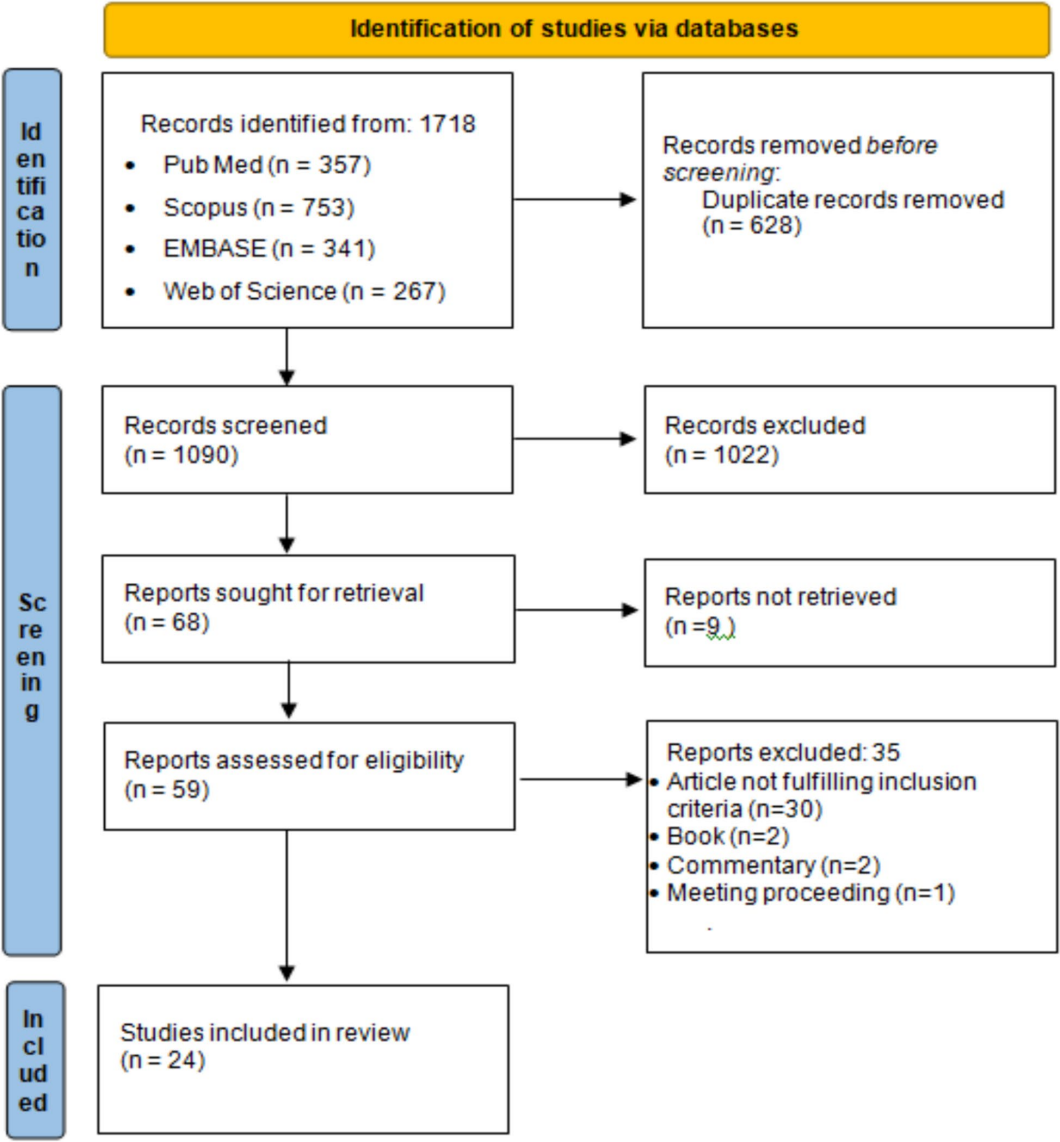
PRISMA-ScR flowchart of study selection

On August 22, 2024, the fundamental literature search was conducted. The search approach is summarized entirely in Additional File 1. Following a thorough search, 1718 articles were found and retrieved; 628 were deemed duplicates. Titles and abstracts were analyzed to filter a total of 1090 articles. Twenty-four of the 68 articles that met the full-text requirements were included in the final review [4, 34–57]. The filtering and selection process results are summarized in the PRISMA-ScR flowchart (Fig. 2).

### Characteristics of the included studies

The major characteristics of the selected studies are shown in Table 2. The majority of the articles chosen were published after 2015 (34–46;48–51;53–57). A review on the “Scientific and Regulatory Challenges for Follow-on Products” by Nicholas in 2012 [47] and “Clinical development, immunogenicity, and interchangeability of follow-on complex drugs” by the same authors in 2014 [51] shed light on follow-up products or otherwise called complex generics. Majority of the included studies focused on regulated markets such as European Union (*n* = 10) (34–37;39–40,43,48,50,54) and the USA (*n* = 8) [41, 42, 45, 47, 51, 52, 54, 56]. India stands next to these regulated markets with two articles (*n* = 2) [4, 55]. Other countries, such as Russia [38], Taiwan [44], China [46], and Malaysia [50], contribute one paper each (*n* = 1 each). In terms of the study design, most papers were review articles (*n* = 17) [4, 34–36, 38–40, 43–48, 50–52, 54, 55], followed by research studies (*n* = 6) [37, 41, 42, 49, 53, 56]. The included papers are more focus on complex formulation (*n* = 13) [4, 34–37, 39, 40, 42, 44, 46, 50, 51, 55], followed by complex formulation in combination with complex API (*n* = 8) [4, 35, 37, 44, 46–48, 52], complex drug device combination product (*n* = 6) [4, 41, 45, 52, 54, 56], complex API (*n* = 2) [43, 44], and complex formulation in combination with complex route of administration (RoA) (*n* = 2) [37, 46]. One paper each highlights the challenges of complex RoA [4] and complex excipients [38].The data from the included studies were divided into main groups of challenges: formulation challenges, analytical challenges, clinical challenges, critical process parameter-based challenges (CPPs), critical quality attribute-based challenges (CQA), regulatory challenges, and other miscellaneous.

**Table 2:** Summary characteristics of the included studies

First author (year) (cite)	Country	Aim	Class of complex generics	Sub-class	Type of challenge	Strategies used to overcome
Demetzos C (2020) [34]	Greece	To investigate the parameters that the scientists and the regulatory authorities should take into account in order to build up a dynamic regulatory landscape for nanomedicines	Complex formulation	Nanomedicine	Formulation Analytical Clinical CPP CQA Regulatory	• SAXS- structural similarities and cryoTEM- morphology and PC • Astrolab (Cross check regulatory implication)
Crommelin DJA (2015) [35]	Netherlands	To show that the situation between biologicals and non-biological complex drugs has important commonalities so to obtain reproducible generic version is challenging	Complex formulation Complex formulation + API Complex formulation + Dosage form	Injectable (glatiramer acetate injection) Injectable (Iron carbohydrate complexes) Nanomedicine (Liposomal Injection)	Formulation Analytical Clinical CPP CQA Regulatory	• Stepwise development approach • Totality of evidence
Arends RJ (2019) [36]	Netherlands	Requires a good understanding of the synthesis process together with a full set of characterization data for establishing therapeutically equivalency	Complex formulation	Injectable (glatiramer acetate)	Formulation Analytical Clinical CQA Regulatory	• Orthogonal bio analytical method-overall mixture properties and batch to batch variability
Hussaarts L (2017) [37]	Netherlands	Focus on assessment of critical attributes to establish equivalence for follow-on versions,	Complex formulation Complex formulation + API Complex formulation + RoA Complex formulation	Injectable (glatiramer acetate) Injectable (Enoxaparin sodium) Cyclosporine ophthalmic emulsions Nanomedicine (Liposomal Injection)	Formulation Analytical Clinical CPP CQA Regulatory	• Orthogonal testing • Develop themes of CQA and physiochemical properties (PC) to biowaive clinical trails
Demina NB (2024) [38]	Russia	A brief overview of complex generics, approaches to their development, and quality control	Complex excipient	Injectables (SandostatinLAR,)	Formulation Analytical Clinical CPP CQA Regulatory	• Extensive tool development research program to identify all CQA and CPP
Rocco P (2019) [39]	Italy	The differences between US and EU regulatory approaches to glatiramer acetate marketing authorization are highlighted	Complex formulation	Injectable (glatiramer acetate)	Formulation Analytical CQA Regulatory	• Orthogonal method
Mühlebach S (2018) [40]	Switzerland	Strategies to overcome the challenges to support progress toward a defined and harmonized regulatory pathway for nanomedicines and their follow-on versions	Complex formulation	Nanomedicine	Formulation Analytical Clinical CPP CQA Regulatory	• Extensive PC quality assessment with appropriate statistical analysis
Choi SH (2018) [41]	US	Providing recommendations on the types of studies necessary to establish BE, on product quality and performance related considerations as well as tools to assess the proposed user interface	Complex DDC	Topical & Transdermal Intrauterine devices Injectables (prefilled syringes) Nasal products Inhalations	Formulation Analytical Clinical CPP CQA	• Pre-ANDA meeting
Astier A (2017) [42]	US	Provides a tool for rational decision making for the inclusion of nanomedicines into the hospital formulary, including defined criteria for evaluation of substitutability or interchangeability	Complex formulation	Nanomedicine	Formulation Analytical Clinical CQA Regulatory	• Tool to evaluate therapeutic equivalence (TE) based on clinical date by pharmacist
Klein K (2019) [43]	Netherlands	Highlight the heterogeneity in the regulatory approach taken for many NBCD follow-on products	Complex API All class of complex NBCD	Sevelamer carbonate (Low molecular weight heparin)	Formulation Analytical Clinical CPP Regulatory	• Use of de-centralized procedure
Liu YH (2023) [44]	Taiwan	Compare the regulatory requirements for developing generic NBCD in EU and US and shown harmonization of the regulatory requirements is necessary	Complex formulation Complex formulation + API Complex API	Nanoparticle albumin-bound paclitaxel injections Liposomal injections Glatiramer acetate injections Iron carbohydrate complexes Sevelamer oral dosage forms	Clinical CPP CQA Regulatory	• Use of product-specific guideline
Newman B (2020) [45]	US	Covers the various aspects of OIDP complexity, address the challenges, and complex drug development	Complex DDC	Inhalers	Formulation Analytical Clinical CPP CQA Regulatory	• Weight of evidence approach Pre –ANDA annual public workshop by ORS (OGD)
Sun Z (2020) [46]	China	Highlight pharmaceutical properties of NBCD pose challenges to interchangeability evaluation of the follow‑on versions and also interpret the currently available guidelines and alternatives to overcome the challenges	Complex formulation Complex formulation + RoA Complex formulation + API Complex formulation + API	Injectables Ophthalmic liposomal drug Parentrals (liposomal or protein-bound) Iron-carbohydrate complexes	Formulation Analytical Clinical CPP CQA Regulatory	• PK-PE study was done in case any difference in qualitative and quantitative sameness
Nicholas JM (2012) [47]	US	Explain the scientific challenges associated with approval of follow-on versions of NBCD	Complex API	Galitatmer acetate Peptide	Formulation Analytical Clinical CPP CQA Regulatory	• ANDA can differencing inactive ingredient and container closure system
Rocco P (2019) [48]	Italy	Analyze the different regulatory approaches to NBCD and their copies in the USA and the EU	Complex API	Glatiramoids	Analytical Regulatory	• Quality by design
Kumar L (2015) [4]	India	The global generic segment, market growth, development, commercialization strategies, and opportunities for innovation in the generic market to develop super generic	Complex API DDC Complex formulation Complex RoA	Absorica™ (Isotretinoin) capsules Dymista Docefrez injection Intravail Tech	CQA	• Modernize QbD
Jauch D (2015) [49]	Germany	Non-tariff barriers to trade have a negative effect on generics exporters	General complex generics	Injectable, respiratory products, patches	Regulatory	• Single development through trade negotiation
Lim YW (2022) [50]	Malaysia	Challenges faced in developing PLGA-based long-acting injectable/implantable (LAI) drug products; hurdles that are associated with drug loading and release and approaches to overcome	Complex formulation	Injectable	Formulation Analytical Clinical CPP CQA Regulatory	• Risk evaluation and mitigation method • Microfluidics and electro spraying method • QronoMetrics- develop LAI
Nicholas JM (2014) [51]	USA	Development of follow-on versions of NBCD poses many of the same scientific challenges associated with biosimilar drugs	Complex formulation Complex formulation + API Complex formulation + API	Liposomes Iron-carbohydrate complexes Glatiramoids	Formulation Analytical Clinical CPP CQA Regulatory	• Assess immunologic and immunogenic safety comparison studies
Walenga RL (2019) [52]	USA	Examines in silico models that may be used to support the development of generic orally inhaled drug products and how model credibility may be assessed	DDC	Inhalers	Formulation Analytical CPP CQA	• Weight of evidence (in vito + in vivo + formulation sameness + device similarities)
Barei F (2015) [53]	France	To investigate whether the use of the term generic would reduce product liability in marketing super generic or hybrid pharmaceuticals, decreased promotion	General	All classes	Regulatory	• QbD approach, use of muti-functional excipients, modifying dosage form and reforming release pattern
Mohan AR (2022) [54]	US	The current understanding of the formulation and device-related principles driving DPI performance, past, and present research efforts to characterize BE	DDC	Inhaler	Formulation Analytical Clinical CPP CQA Regulatory	• Use of multifunctional excipients
Paliwal R (2022) [55]	India	Summarized the silent features of the regulatory perspectives related to nanotechnology based next generation therapeutics and diagnostics	Complex formulation	Nanomedicine	Formulation Analytical Clinical CPP CQA Regulatory	• Early engagement with regulatory agency • More focus on chemistry, manufacture and control (CMC) parameter
Feldman WB (2023) [56]	US	To compare the effectiveness and safety of Wixela Inhub and Advair Diskus among patients with COPD	DDC	Inhaler	Clinical Regulatory	• Weight of evidence approach

#### Formulation challenges

Complex generics present highly complicated challenges because of their heterogeneous nanostructures, complex manufacturing processes, and sophisticated analytical or characterization techniques. To address these challenges, an effective, controlled manufacturing technique is essential to ensure efficacy, safety, and quality [34]. The major hurdle is that existing physicochemical analytical methods are unable to characterize and detect structures completely, especially those present in non-biological complex medicines (NBCD) [40]. In addition, changes in product availability and deposition cause hemosiderosis and oxidative and nitrosative stress, highlighting the strict implementation of stepwise manufacturing procedures and the totality of the evidence approach [35]. Batch-to-batch heterogeneity tends to be challenging when considering the demonstration of bioequivalence at the substance level of each complex generic, especially glatiramer acetate (complex API) [36]. For example, accurate physiochemical full-set characterization of complex generics such as Copaxone and liposomal doxorubicin cannot be performed, so biophysical characterization profiling needs to be employed as an advanced characterization technique [37]. Differences in the properties of inactive ingredients, such as their molecular weights, copolymer component ratios, and polymer branching, affect their pharmacokinetic properties, release profiles, and the effectiveness of their dosage forms [38]. To enhance performance, advancements such as the merging of poly lactic-glycolic acid (PLGA) with additional polymers are needed because PLGA formulations frequently show quick release at first, preceding slow and minimal release [50]. Transdermal delivery systems constitute one of the most advanced and challenging classes of commercially available drug‒device combination products. Variation in the design, adhesiveness, selection of excipients, and manufacturing controls leads to leakage, difficulty in removal, residual drug deposition on the skin, and, ultimately, the performance of the TDS, which directly affects the consistency and therapeutic efficacy [41]. The surface area coverage (SAC) and aerodynamic particle size distribution (APSD) are the critical formulation parameters that affect the performance of orally inhaled and nasal drug products (OIDPs). The impact of excipients also affects OIDPs [52, 54]. The variation in the cosolvent concentration in pressurized metered-dose inhalers (MDIs) determines their droplet size and significantly impacts their performance [45]. Detailed formulation selection criteria, including formulation performance, manufacturing considerations, safety, effectiveness, and patient-specific attributes, must be considered in regard to nanosimilars [42, 55]. The physicochemical characteristics of NBCD, such as their solubility, stability, and physiological sensitivity, make it even more challenging to assess interchangeability [46]. Current technologies cannot fully identify the various potential epitopes present in heterogeneous mixtures (glatiramer acetate). Immunogenic pharmaceutical modifications might raise serious safety issues, highlighting the necessity of strict control over production conditions [47]. Complex generics present a variety of formulation constraints that necessitate an in-depth understanding of their physicochemical, formulation, analytical, and regulatory aspects.

#### Analytical challenges

The primary developmental hurdle in the case of complex generics is the inability to establish bioequivalence via conventional analytical methods [37]. Therapeutic equivalence (TE) can be shown from pharmaceutical and bioequivalence data, but it is more complicated compared with other methods [47]. For example, excipients used in liposomal dosage forms must undergo stability testing of vesicles, compatibility with drugs, excipients, and other inactive ingredients that significantly affect the size, surface coating, polydispersity index, and in vitro as well as in vivo stability of the nanoparticles [55]. Moreover, the colloidal forces highlight the need for sophisticated tools to assess their dynamic physicochemical interactions in various solvents [34]. The multifaceted deposition patterns of NBCD are frequently ignored by traditional BE studies, which depend on pharmacokinetic (PK) parameters, including the volume of distribution (Vd) and clearance (Cl). The tissue-specific drug distribution and physiochemical properties, such as particle size, surface morphology, particle distribution, encapsulation stability, and release kinetics, are challenging to characterize via noncompartmental studies [46]. The limits of current methods in guaranteeing repeatability and therapeutic equivalency are also highlighted by the batch-to-batch variability shown in drugs such as Copaxone [36, 39, 51]. Equivalence evaluations are made more difficult by analytical testing, which reveals differences in gene transcription sequences when splenocytes from experimental mice are triggered with glatiramer acetate in multiple batches [51]. Most of the following versions of NBCD are nanostructures or nanomedicines with heterogeneous structures, so they face unique challenges in establishing their TE [40, 42, 51]. Currently, API analysis requires a standard methodology that also determines the structure‒activity relationship of an API with a copolymer because the structural architecture of the polymer varies widely (e.g., linear PLGA, star-shaped PLGA, and branched PLGA) [38]. In the case of the biological complex API, the BE is not enough to establish the TE because of the variation in the pharmacodynamic profiles [48]. The in vitro release results of a PLGA-based complex formulation are variable when the conventional method is tested. All these challenges necessitate a new compendia in vitro release testing (IVRT) method to ensure the reproducibility and discrimination of different batches of PLGA-based complex generics [50]. In the case of drug‒device combinations, minor variations in device design (pressure drop, spray velocity, particle size, velocity, and residual time) [54] and drug-related issues (API solubility and drug deposition in the lungs) [45] significantly affect the characterization stage (mainly in the case of inhalers) [52]. The transdermal dosage form is another challenging class of drug‒device combination complex generic in which the heat effect causes leakage of the dosage form and cold flow during storage [41]. These parameters underscore the need for advanced analytical techniques for all classes of complex generics. Delays in identifying and sorting these analytical challenges adversely affect the safety and efficacy of profiling [43].

#### Clinical challenges

Nonbiological follow-up products, nanosimilars, and generic drug‒device combination products pose significant challenges in proving their safety and efficacy [44, 47]. The therapeutic equivalence of heterogeneous molecules as well as TDS can be established via comparative studies (physiochemical and pharmacokinetic), nonclinical assessment techniques (in vitro and in vivo), and comparative clinical studies to address associated clinical challenges [36, 41]. In the case of certain complex formulations, such as liposomal complex generics, the difficulties are not only because of heterogeneity but also coupled with dynamic changes within the biological environment. Because of these hurdles, complex formulations require a more rigorous and sequential clinical assessment plan to ensure safety and efficacy [34]. Unpredicted drug release and in vivo deposition are the most challenging factors in clinical assessment because of the critical influence of dissociation kinetics between the drug and its carrier [37]. In the case of microspheres, factors such as the glass transition temperature and porosity alter the drug release rate, which leads to a potential deviation in therapeutic efficacy [50]. Moreover, comparative clinical endpoint BE studies are mostly less sensitive for identifying formulation differences because they rely more on patient variability [45]. Corticosteroidal inhalation has shown the aforementioned problem, where demonstrating a dose‒response relationship has been difficult owing to deviations in patient response and the dynamics of drug deposition in the lungs [45]. Determining nanomedicines’ structure‒activity relationships (SARs) is essential for chemists and other healthcare professionals to guarantee safe and efficient usage. By using this strategy, patient variability issues can be minimized [42, 55]. The immunogenicity of complex generics is another critical hurdle that comes across the clinical assessment stage. In the case of iron sucrose follow-up products, deviation in formulation-related labile iron release affects both efficacy and toxicity [46]. Protein/peptide aggregation or impurities formed during synthesis may accelerate immunogenic responses and increase the likelihood of undesirable effects. Comparative immunogenicity studies are needed to evaluate possible differences between the reference product and its subsequent variants [51]. Generic versions of complex drugs such as doxorubicin HCl, daunorubicin citrate, and amphotericin B highlight the variation in the immunogenic response concerning formulation parameters [46]. Regulatory concerns further complicate the clinical safety assessment of complex generics [40]. A thorough phase III clinical trial is frequently necessary for complex generics. Considering its heterogeneous character, glatiramer acetate follow-up products in Europe need a thorough clinical evaluation (phase III) before being authorized for sale [36]. Demonstrating therapeutic equivalency is challenging owing to several factors, including patient variability, immunogenicity threats, regulatory obstacles, and the complicated nature of drug release. Unique administrative procedures and designs are the main factors that cause clinical variations in drug device combination products, especially DPI.

#### Critical process parameter and critical quality attribute-based challenges.

**Fig. 3:**
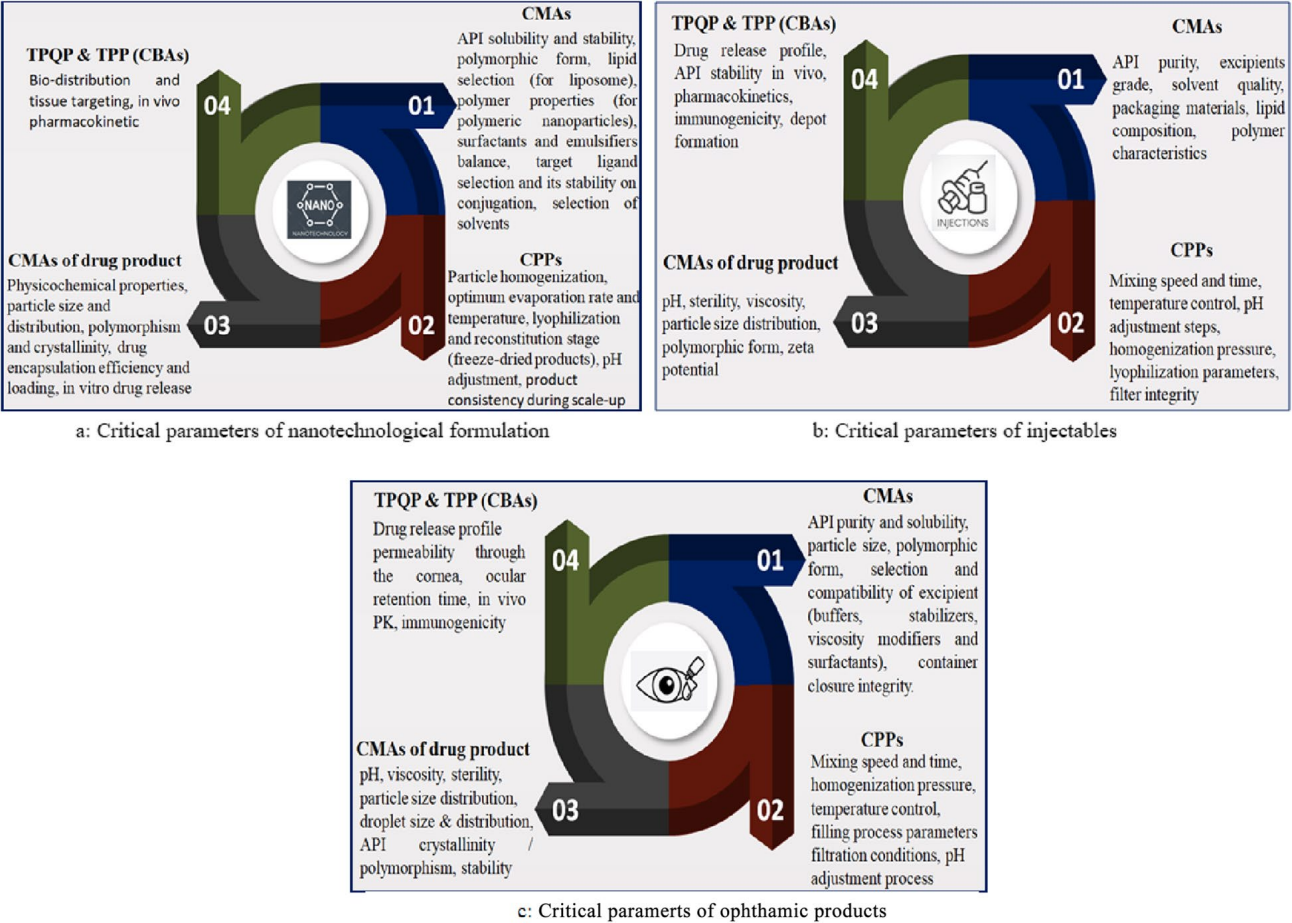
The overall critical parameters related to the main class of complex generics such as nanotechnological, injectables and occular products

CPP- and CQA-based challenges include challenges related to critical material attributes (CMAs) of active pharmaceutical ingredients, CMAs of excipients (both functional and nonfunctional), critical formulation parameters, CMAs of the formulated dosage form, and critical bioavailability parameters (target product performance quality profiling parameters and target product efficacy profiling) [4, 34–56]. The overall CQAs and CPPs are depicted in Fig. 3a (nanotechnological formulation), Fig. 3b (injectable formulation), and Fig. 3c (ophthalmic formulation).

#### Regulatory challenges

Complex generics need a more comprehensive approval process than typical small-molecule generic medicines do, which frequently include comparative clinical trials, in vitro and in vivo nonclinical research, and physicochemical characterization [36, 44]. Regulators and prospective applicants are concerned about the current regulatory paths because of the lack of clarity and uniformity [40]. The absence of a coordinated strategy for NBCD and nanomedicines is one of the major regulatory obstacles. For example, similar NBCD submissions might be assessed under a decentralized system, resulting in inconsistent regulatory standards, whereas biosimilars in the EU follow a centralized approval process [35]. In the USA, NBCD follow-ons are regarded as generic drugs, whereas in the EU, they are a unique class of drugs known as hybrid medicines that are approved by specialized national agencies [48]. For example, applicants find it challenging to conduct suitable bioequivalence studies owing to the restricted number of product-specific guidelines (PSGs) that exist for long-acting injectable (LAI) medications on the basis of poly(lactic-co-glycolic acid) (PLGA) [50]. Because present guidelines do not often offer precise recommendations, regulatory agencies also encounter difficulties in identifying the criteria and analytical methods that are appropriate for evaluating subsequent NBCD [38]. An alternative regulatory classification for NBCD has not been established by the FDA or European regulators. To expedite approval procedures and prevent unnecessary repetition of comparative clinical research, regulatory consistency across areas is essential [39, 43, 45]. Currently, to satisfy region-specific regulations, applicants seeking marketing authorization in both the USA and the EU frequently have to carry out independent clinical trial programs [49, 55]. Delays in accessibility for patients, higher expenses, and ethical issues with unnecessary testing result from this. Complex generics’ market entry techniques have also drawn attention; some businesses are promoting follow-up versions such as “super generics” or “value-added generics,” which could result in reimbursement obstacles [53]. It is crucial to assess the suitability of the present regulatory systems in light of the growing complexity of innovative treatment interventions [37]. The main regulatory issues are examined in this scoping assessment, such as inconsistent approval processes, a lack of product-specific guidance, and the effect of regulatory variations on market access [51].

#### Miscellaneous challenges

The lack of internationally accepted classification of complex generics is one of the most challenging factors [55]. For example, the following versions of low-molecular-weight heparins are considered complex generics in the USA, whereas in the EU, they are considered biosimilars [43]. This discrepancy in the classification of complex generics negatively affects their market authorization. Multidisciplinary efforts are needed to overcome multidisciplinary challenges and establish equivalence for the NBCD generic version [36]. Additionally, citizen petitions and restricted access to branded drugs limit the market entry of cost-effective complex generics [50, 53]. In the case of drug device combination (DDC), even though the patent expires for the drug, it is challenging to develop a generic version because of the availability of separate design patents for the device interface [52]. When looking into the market dynamics of complex generics, these products took 5–6 years for market entry [4]. The main reason is the regulatory requirement of clinical endpoint studies, where recruiting patients as research participants rather than healthy volunteers for BE studies takes more time [46]. Furthermore, the complicated intellectual property (IP) regulations in a few countries, such as Canada, make it more difficult for generic producers to enter new markets [49]. Investments in development and, at the same time, the supply of cost-effective medication are other major challenges, especially in the case of nanosimilars [34]. Simpler and more affordable alterations can have an impact on the quality of drugs [38]. Some rare challenges are found mainly for complex APIs, such as the following version of copaxone (probioglat), which has shown variation in its transcriptional profile [35], and an incomplete elucidation of its mechanism of action [47].

All identified challenges are summarized in Table 3. The parameters that are highlighted in this review such as particle size distribution, surface morphology, encapsulation efficiency, release kinetics, aerodynamic characteristics, and device interface were selected because of their broad prevalence and significance among the literature. For complex generics, these factors are essential to establishing bioequivalence and attaining reliable therapeutic effects. Although primary testing was not conducted by us, the included studies showed that these parameters were very sensitive to formulation and process conditions. For instance, excipient grade and device design varies and altered aerosol performance in inhalers [52, 54], while polymer porosity and composition varied widely and drastically affected drug release in long-acting injectables [50]. These findings emphasize the necessity of rigorous regulation of these characteristics, which explains why they are given highest priority in our synthesis.

**Table 3:** A summary of different types of challenges

Type of challenges	Description
Formulation	Heterogeneous nanostructures of NBCD Sophisticated manufacturing methods and process controls Batch to batch heterogeneity Differences in the properties of inactive ingredients/excipients Issues based on route of delivery and the advanced formulation Variation in drug content and release kinetics of heterogeneous NBCD Variation in the design and adhesiveness (in case of TDS) Challenges due to immunogenic pharmaceutical modifications
Analytical	Difficulty to ensure similarity using conventional BE approaches Difficulty to ensure equivalence in particle size, its distribution, encapsulation efficiency, release kinetics, and other parameters Batch to batch variation in different analytical parameters Limitation to PK studies Need for advanced technologies live orthogonal, IVRT, biophysical, and so on Physicochemical analytical methods are unable to characterize and detect structures completely
Clinical	Variation in patient response (mainly in DPI) Dynamic interaction between drug and carrier Variation in endpoint BE study Immunogenicity risk (mainly in complex API) Need for phase III clinical study reports Lack of TPP and TPQA Lack of standardized list of physiochemical characteristics required
CPP & CQA	CMA and drug and excipients CPP and their controls affecting BE Variations in CMAs, CPPs, CBAs of drug formulation Selection of excipients and manufacturing controls
Regulatory	Lack of harmonized guidelines Wide variation in regional approval steps Lack and concurrent revisions in PSG Reimbursement and labeling barriers Need for phase III clinical study reports
Miscellaneous	Citizen petition market exclusivity challenges Huge investment and time Lack of proper definition Stakeholder challenges Supply chain challenges

## Discussion

There are prospects in a new area of the pharmaceutical industry that are driven by the complexity of complex generics. Quality by design (QbD) is one of the foundations that may help the development of high-quality complex generics in a shorter time. This method thoroughly examines and minimizes the product design phase time, variability in quality characteristics, review period, risk, and regulatory uncertainty [57]. While continuously producing high-quality drugs, the updated method enables producers to optimize the economic feasibility of the product. Market leaders can effectively deliver value-added medicines through the use of multifunctional excipients, modifications to dosage forms, and reworking of drug release patterns [4]. In addition to improving results, these advancements have opened profitable markets and advanced the healthcare sector’s competitive picture. The FDA implemented QbD as a positive strategy to increase the number of high-quality ANDA of complex generics in 2013 [58]. Certain projects related to the identification of critical quality attributes of dry powder inhalers are now utilizing the QbD approach [45, 59].

The US government supports several targeted programs and provides financial support. This fund was provided in 2020 by the US FDA to the University of Michigan and the University of Maryland to establish an organization to increase collaboration as well as interaction among academia, researchers, industries, and regulatory bodies. With this goal, both universities establish CRCG [38, 60]. Over 100 extramural projects are awarded to the Office of Generic Drugs, which encompasses nine projects related to the challenges faced during the development and manufacturing of generic DDC products [41]. The NBCD working group provides a platform for all stakeholders to discuss equivalency-related challenges and findings to develop a science-based method and establish harmonized guidelines [40, 61]. Additionally, these collaborative initiatives/strategies highlight how important pre-ANDA meetings are for informing applicants of regulatory requirements early in the development cycle [62]. Frequent workshops have made it easier for industry stakeholders and authorities to communicate. These workshops address critical issues faced by generic applicants and provide insights into the agency’s expectations for demonstrating therapeutic equivalence in ANDA [44].

Technological innovations also pave the way for the development of cost-effective generic versions of complex drugs. These tools, such as in silico models, help develop generic orally inhaled drug products. Using this model to simulate drug delivery systems and evaluate their performance, such as by predicting the spray angle, regional deposition, velocity, and other characteristics, enhances the credibility of the product [52]. Molecular modeling further describes small-molecule drug‒biopolymer interactions, mainly in the case of PLGA and small-molecule drugs [50, 63].

Collaborative efforts can substantially boost the progress made in complex generics. An example of such successful collaboration is between Dr. Reddy’s Laboratories of India and the US-based company Aegis Therapeutics, which have developed excipients exhibiting highly systemic bioavailability. These innovations are equivalent to injectable formulations and provide the means for delivering potent peptides, proteins, and large molecules, thus offering new therapeutic options for patients via the intranasal or transmucosal route [64]. The International Generic and Biosimilar Medicines Association remains an influential body in building global collaborations and market development by stimulating information exchange and advocacy for the global acceptance of generic and biosimilar medicines [49, 65]. In addition, the seamless implementation of risk evaluation and mitigation strategies also counteracts supply chain limitations, such as those facing resident raw material supplies for manufacturing generic medicines [50, 66].

In addition, introducing alternative biomarkers into the regulatory framework facilitates the approval process for complex generics. This new trend allows respective agencies to scrutinize therapeutic equivalence rather than just bioequivalence, leading to a more rational stand concerning interchangeability and substitutability [45]. This new framework ensures effective and reliable patient treatment by increasing the barriers to and impediments to generic market entry. In addition from the data sheet, we can conclude the overall initiatives or methods used to overcome or the identified challenges depicted in Table 4. Complex generics are likely to provide affordable, high-quality medicines in parts of the world with dire conditions that should otherwise spur hopes for healthier people by saving lives.

**Table 4:** Initiatives or methods used to address the challenges

S. No	Focused area	Methods to overcome
1	Regulatory initiatives	Tool for cross-checking real-time implementation: Regulatory astrolabe Ensure clarity in design and approval expectations: PSG Harmonization in guidance is upcoming. e**.**g., “EMA reflection paper on iron based nanocolloids” Emphasize on Pre-ANDA meetings and use of control correspondence Emphasize more on the use of weight-of-evidence approach
2	Advanced methods for demonstrating bioequivalence	To minimize variability and define parameters/controls: **i**mplement QbD To identify CQAs: validated in vitro/in vivo models Study the effects of PK variables based on excipients Use of both in vitro and in vivo study (case of DPI and MDI) Use of comparative biological assay/immunogenicity study to prove the API sameness
3	Intellectual property	Use of combined guidance to improve the quality of data submission. e.g., US (505B(2) + 505 J); EU (Hybrid application**-** Article 10(3)) Use Risk Evaluation and Mitigation Strategies
4	Technological advances	*Liposomal products*: structural similarity (small angle X-ray scattering); morphological and PC characterization **(**Cryo-TEM**)** *Complex API*: evaluate batch-to-batch variability and overall mixture properties (orthogonal bioanalytical methods) *LAI*: **e**valuate polymer interaction (Molecular modeling tools) *DPI:* reduce particle agglomeration (using coating techniques)
5	Educational initiatives	FDA establish CRCG to support research Collaboration between IGBA to promoting growth and exchange of information globally

## Strengths and limitations

The strength of the current study lies in providing a thorough summary of the strategies used by various stakeholders to address the challenges encountered during development until the patient is reached. It also provides an overview of the innovative strategies used to bridge the knowledge gap in the selected disciplines. The rise of recent articles on complex generics indicates that this field is expanding quickly. This exploratory scoping review covered various sources, challenges, and countries. This evaluation captured a comprehensive view of the available literature, including the articles and review articles extracted from the databases. One of the main obstacles to identifying the best challenges was the diversity of the literature. The database analysis was performed until December 2024, and more recent research in this area may have been released; thus, this review provides in-depth knowledge of the literature. The purpose of a scoping review is to outline and summarize the available evidence; it is not necessary to evaluate the research methods; hence, quality evaluations of the included publications were not carried out [33]. The publication of solely favorable results could add potential bias. However, there might be some restrictions. For example, the information gathered from a few databases did not include information on strategies that have been proven effective and approved by regulatory bodies; instead, it focused on strategies that were used at the time or for a particular product by a particular group of stakeholders from a specific industry. The research studies selected have no framework and exhibit significant differences within the same class of complex generics. Although potentially pertinent research conducted in other languages was not included in this scoping review because the selection criteria were restricted to English-language papers, this is anticipated to have a negligible effect on the results. The primary sources of the scientific databases used were PubMed, EMBASE, Scopus, and Web of Science. Furthermore, despite the size of the databases, access to other potentially pertinent databases may have been restricted, leading to their exclusion.

## Conclusion

The difficulties of different classes of complex generics underscore the necessity of scientific breakthroughs, streamlined analytical testing, and an integrated regulatory strategy. However, regional regulatory disparities, such as the different approval processes in the USA and Europe, pose serious challenges for manufacturers, increasing expenses and delaying patient access. The lack of clear regulatory groupings for complex generics, disparate data requirements among agencies, a lack of product-specific guidance for bioequivalence studies, and a lack of standardized techniques for evaluating physicochemical properties, pharmacokinetics, and immunogenicity are some of the major obstacles. Increased global collaboration and regulatory harmonization are necessary to overcome these problems. Approval can be expedited while maintaining patient safety by setting precise standards for assessing complex generics, such as standardized bioequivalence and therapeutic equivalency criteria. Implementing a risk-based, science-driven strategy will improve market access for affordable substitutes for branded drugs and streamline regulatory decision-making. In the end, removing these regulatory obstacles will lower healthcare expenses, promote innovation in medication research, and increase patient access to high-quality complex generics. The goal of this study is to offer information that will assist in making approval procedures for complex generics more effective and transparent.

## Supplementary information

Additional file 1: Preferred Reporting Items for Systematic Reviews and Meta-Analyses extension for Scoping Reviews (PRISMA-ScR) checklist.

Additional file 2: Search queries and strategies via electronic databases.

Additional file 3: Studies included in the data analysis.

## Data Availability

The datasets used and/or analyzed in the present study are available from the corresponding author upon reasonable request.

## Abbreviations

PRISMA-ScR: Preferred Reporting Items for the Scoping Reviews Extension for Systematic Reviews and Meta-Analyses
ANDA: Abbreviated New Drug Application
FDA: Food and Drug Administration
BE: Bioequivalence
EMA: European Medicines Agency
IVIVC: In vitro in vivo correlation
TDS: Transdermal delivery system
CRCG: Center for Research on Complex Generics
IVR: In vitro release
PCC: Problem, concept, and context
MeSH: Medical Subject Headings
CPP: Critical process parameter
CQA: Critical quality attribute
PC: Physiochemical properties
TE: Therapeutic equivalence
API: Active pharmaceutical ingredients
NBCD: Non-biological complex medicines
OIDPS: Orally inhaled and nasal drug products
DDC: Drug device combination
RoA: Route of administration
QbD: Quality by design
LAI: Long-acting injectable/implantable
CMC: Chemistry, manufacture, and control
PLGA: Poly lactic-glycolic acid
SAC: Surface area coverage
APSD: Aerodynamic particle size distribution
MDI: Metered-dose inhalers
PK: Pharmacokinetic
Vd: Volume of distribution
Cl: Clearance
IVRT: In vitro release testing
SAR: Structure‒activity relationships
PSG: Product-specific guidelines
