# Supplementary material for: Exploring the challenges faced by generic version of complex drugs: a scoping review
Source: Syst Rev. 2025 Sep 29;14:183. doi: 10.1186/s13643-025-02931-y (PMC12482112; doi:10.1186/s13643-025-02931-y)
Supplement: Supplementary file 2 — Additional file 2: Search queries and strategies via electronic databases. [file 13643_2025_2931_MOESM2_ESM.pdf]

S.no	Database	Filters	Search Details
1	Pubmed	Language- English	("complex drug"[Text Word] OR "Complex generic"[Text Word] OR "Hybrid medicines"[Text Word] OR "Super generic"[Text Word] OR "non-biological complex drugs"[Text Word] OR "drug device combination"[Text Word]) AND ("challenge"[All Fields] OR "challenged"[All Fields] OR "challenges"[All Fields] OR "challenging"[All Fields] OR ("opportune"[All Fields] OR "opportunities"[All Fields] OR "opportunity"[All Fields]))
2	Scopus	Language- English	(TITLE-ABS-KEY("complex drug") OR TITLE-ABS-KEY("Complex generic") OR TITLE-ABS-KEY("Hybrid medicines") OR TITLE-ABS-KEY("Super generic") OR TITLE-ABS-KEY("non-biological complex drugs") OR TITLE-ABS-KEY("drug device combination")) AND (ALL(challenge) OR ALL(challenged) OR ALL(challenges) OR ALL(challenging) OR (ALL(opportune) OR ALL(opportunities) OR ALL(opportunity)))
3	Embase (Elseiver)	Language- English	('complex drug' OR 'Complex generic' OR 'Hybrid medicines' OR 'Super generic' OR 'non-biological complex drugs' OR 'drug device combination') AND (challenge OR challenged OR challenges OR challenging OR (opportune OR opportunities OR opportunity))
4	Web of Science (Advance)	Language- English	(ALL="complex drug" OR ALL="Complex generic" OR ALL="Hybrid medicines" OR ALL="Super generic" OR ALL="non-biological complex drugs" OR ALL="drug device combination") AND (ALL=challenge OR ALL=challenged OR ALL=challenges OR ALL=challenging OR (ALL=opportune OR ALL=opportunities OR ALL=opportunity))

Results

357

753

341

267
